# Genome-wide analysis and functional characterization of *CHYR* gene family associated with abiotic stress tolerance in bread wheat (*Triticum aestivum* L.)

**DOI:** 10.1186/s12870-022-03589-7

**Published:** 2022-04-20

**Authors:** Hao Liu, Wenbo Yang, Xingli Zhao, Guozhang Kang, Na Li, Huawei Xu

**Affiliations:** 1 College of Agriculture, Henan University of Science and Technology, Luoyang, 471000 Henan People’s Republic of China; 2 Cereal Crops Research Institute, Henan Academy of Agricultural Sciences, Zhengzhou, 450046 Henan People’s Republic of China; 3 National Key Laboratory of Wheat and Maize Crop Science, Henan Agricultural University, Zhengzhou, 450046 Henan People’s Republic of China

**Keywords:** CHYR genes, Genome-wide identification, Gene expression, Abiotic stress, Wheat (*Triticum aestivum* L.)

## Abstract

**Background:**

CHY zinc-finger and RING finger (CHYR) proteins have been functionally characterized in plant growth, development and various stress responses. However, the genome-wide analysis was not performed in wheat.

**Results:**

In this study, a total of 18 *TaCHYR* genes were identified in wheat and classified into three groups. All *TaCHYR* genes contained CHY-zinc finger, C3H2C3-type RING finger and zinc ribbon domains, and group III members included 1–3 hemerythrin domains in the N-terminus regions. *TaCHYR* genes in each group shared similar conserved domains distribution. Chromosomal location, synteny and *cis*-elements analysis of *TaCHYR*s were also analyzed. Real-time PCR results indicated that most of selected 9 *TaCHYR* genes exhibited higher expression levels in leaves during wheat seedling stage. All these *TaCHYR* genes were up-regulated after PEG treatment, and these *TaCHYR*s exhibited differential expression patterns in response to salt, cold and heat stress in seedling leaves. The growth of yeast cells expressing *TaCHYR2.1*, *TaCHYR9.2* and *TaCHYR11.1* were inhibited under salt and dehydration stress. Moreover, gene ontology (GO) annotation, protein interaction and miRNA regulatory network of *TaCHYR* genes were analyzed.

**Conclusions:**

These results increase our understanding of *CHYR* genes and provide robust candidate genes for further functional investigations aimed at crop improvement.

**Supplementary Information:**

The online version contains supplementary material available at 10.1186/s12870-022-03589-7.

## Background

Wheat (*Triticum aestivum* L.) is one of the most important grain crops in the world. However, as sessile organism, wheat typically suffers from various adverse conditions including drought, salinity, cold or high temperature during the growth and development periods, which can directly affect the overall wheat production. Therefore, plants have developed complex regulatory mechanisms to avoid or defend against adverse conditions in the long-term evolution process [1]. CHYR protein (CHY zinc-finger and RING finger protein) is one of important stress-responsive protein to respond to abiotic stress in plants [2, 3].

CHYR proteins contain CHY-zinc finger and C3H2C3-type RING finger domains [2]. According to conserved motifs and phylogenetic relationships analysis, CHYR proteins are essentially classified into three groups: group I, II, and III [2]. CHY zinc-finger domain is defined based on its N-terminal conserved amino acid sequences ‘CXHY’, this domain contains many conserved cysteine and histidine residues, which can play roles in physical interaction, ubiquitination and binding to zinc ions [3–6]. C3H2C3-type RING finger domain, also referred to as RING-H2 finger, exists in the C-terminal region of CHYR protein, that can bind two atoms of zinc and may be involved in mediating protein–protein interactions [4, 7]. Besides, group III members, also named as BTS/BTSL (BRUTUS/BRUTUS-like) or HRZ (hemerythrin RING zinc-finger) proteins, which contain N-terminal hemerythrin (HHE) domains and play an essential role in regulating iron homeostasis [8–11].

CHYR proteins, belonging to RING-type E3 ubiquitin-protein ligase, perform vital functions in plant growth, development and various stress responses via protein ubiquitination degradation [3, 8, 12]. *Arabidopsis AtCHYR1* gene can positively regulate stomatal closure and improve drought tolerance via SnRK2.6-mediated phosphorylation [3]. *PeCHYR1*, which was isolated from *Populus euphratica*, also enhances drought tolerance via ABA-induced stomatal closure caused by hydrogen peroxide (H_2_O_2_) production in transgenic poplar plants [13]. However, the homologous gene of *AtCHYR1*, rice (*Oryza sativa*) *OsRZFP34* gene enhances stomatal opening [14]. MIEL1 can interact with transcription factor MYB30 resulting in MYB30 proteasomal degradation and the suppression of *Arabidopsis* defense and hypersensitive responses (HR) [12, 15]. The *MIEL1* also negatively regulates cuticular wax biosynthesis in *Arabidopsis* stems [15]. *BTS* genes play crucial roles in drought stress responses by facilitating the degradation of transcription factors Vascular plant One-Zinc finger 1/2 (VOZ1/2) protein [16]. *BTS* also plays an essential role in regulating iron homeostasis of plants [11, 17].

*CHYR* genes have been identified in diverse plants, such as maize (*Zea mays*), *Arabidopsis*, rice, and soybean (*Glycine max*) [2, 8, 14, 18], however, a genome-wide identification of *CHYR* genes in wheat was not preformed. In this study, a genome-wide analysis of *CHYR* genes was performed in wheat to characterize their sequences, evolutionary relationships, expression patterns and stress tolerance in yeast cells under various abiotic stress treatments. These results will provide a valuable foundation for further functional studies of *TaCHYR* genes under abiotic stress.

## Results

### Characteristics and phylogenetic analysis of *TaCHYR*s in *T. aestivum*

A total of 18 *CHYR* genes, encoding 50 transcripts, were identified from a wheat genome based on a Hidden Markov Model (HMM) search against of a CHY zinc-finger domain (PF05495) (Table S1). According to the chromosome position and phylogenetic relationship of *TaCHYR* genes, we named them from *TaCHYR1* to *TaCHYR18*. These 18 predicted *TaCHYR* genes encoded polypeptides of 245 (*TaCHYR7.2*) to 1245 (*TaCHYR17.8*) amino acid residues with the putative molecular weights ranging from 28.03 to 140.21 kDa. The isoelectric point (*p*I) value varied from 5.59 (*TaCHYR16.5*) to 7.55 (*TaCHYR9.2*), and the calculated grand average of hydrophilic index (GRAVY) was from -0.166 (*TaCHYR6.2* and *TaCHYR6.3*) to -0.484 (*TaCHYR11.2*), suggesting that these 18 *TaCHYR* genes were hydrophilic proteins. In addition, the subcellular localization prediction indicated that TaCHYR proteins were all located in the nucleus.

**Fig. 1:**
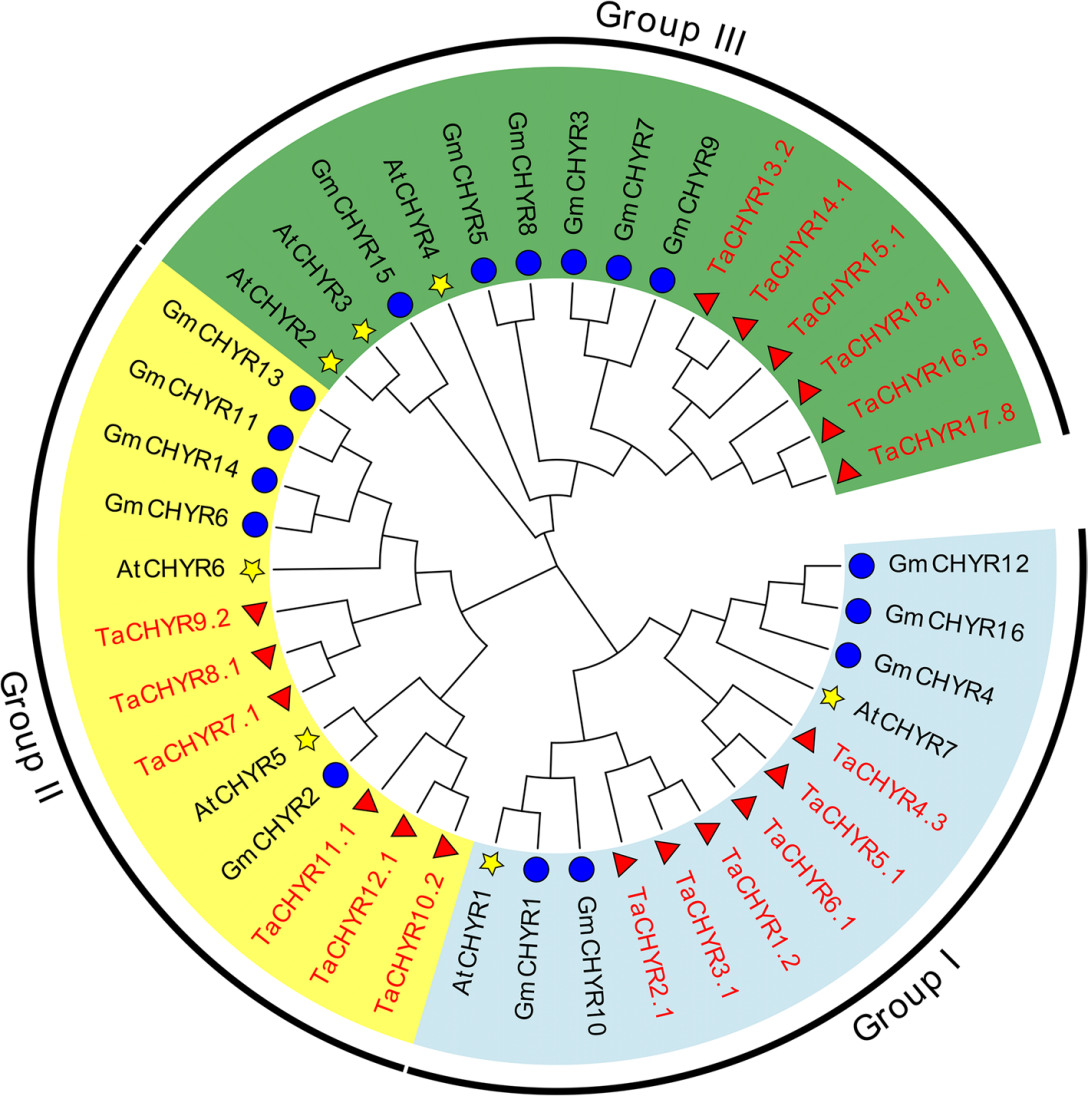
The neighbor-joining (NJ) phylogenetic tree of CHYR proteins. The tree was constructed with protein sequences encoded by the longest transcript of each CHYR gene in *T. aestivum* (Ta), and CHYR proteins sequences in *A. thaliana* (At) and *G. max* (Gm) with bootstrap values of 1000 replicates. Different groups of CHYR proteins were distinguished by different colors

**Fig. 2:**
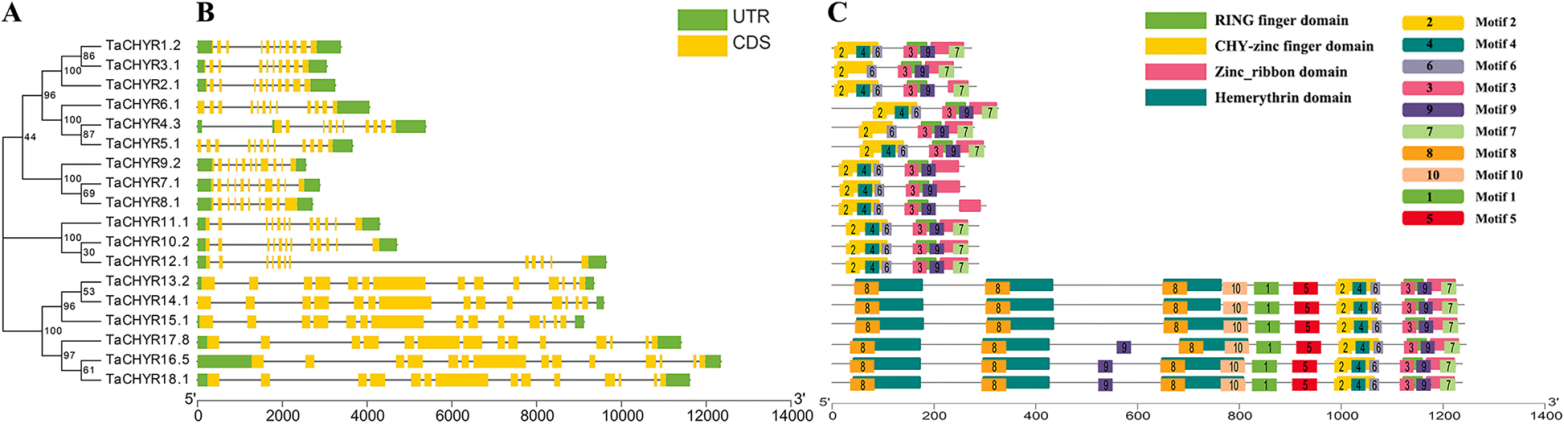
Phylogenetic classification **A**, exon–intron structures **B** and conserved domains **C** analysis of *TaCHYR* genes. **A** The neighbor-joining (NJ) phylogenetic tree was constructed with protein sequences encoded by the longest transcript of each CHYR gene in *T. aestivum* with bootstrap values of 1000 replicates. **B** Green boxes, yellow boxes and black lines indicated UTR, exons and introns, respectively. **C** Conserved domain compositions of TaCHYR proteins in wheat. CHY-zinc finger, C3H2C3-type RING finger, zinc ribbon and hemerythrin domains and motif 1–10 were showed by different colors

To further investigate the phylogenetic relationship of TaCHYRs, AtCHYRs and GmCHYRs were also used to construct a phylogenetic tree (Fig. 1 and Table S2), the results indicated that *TaCHYR* genes were divided into 3 subfamilies: group I, II, and III (Fig. 1 and Fig. 2A), which was consistent with previous phylogenetic analyses of plant *CHYR* genes [2]. Group I (*TaCHYR1*-*6*), group II (*TaCHYR7*-*12*) and group III (*TaCHYR13*-*18*) subfamilies included 6 members in wheat, respectively (Fig. 1 and Table S1).

### Gene structure and conserved motifs analysis of *TaCHYR* genes

**Fig. 3:**
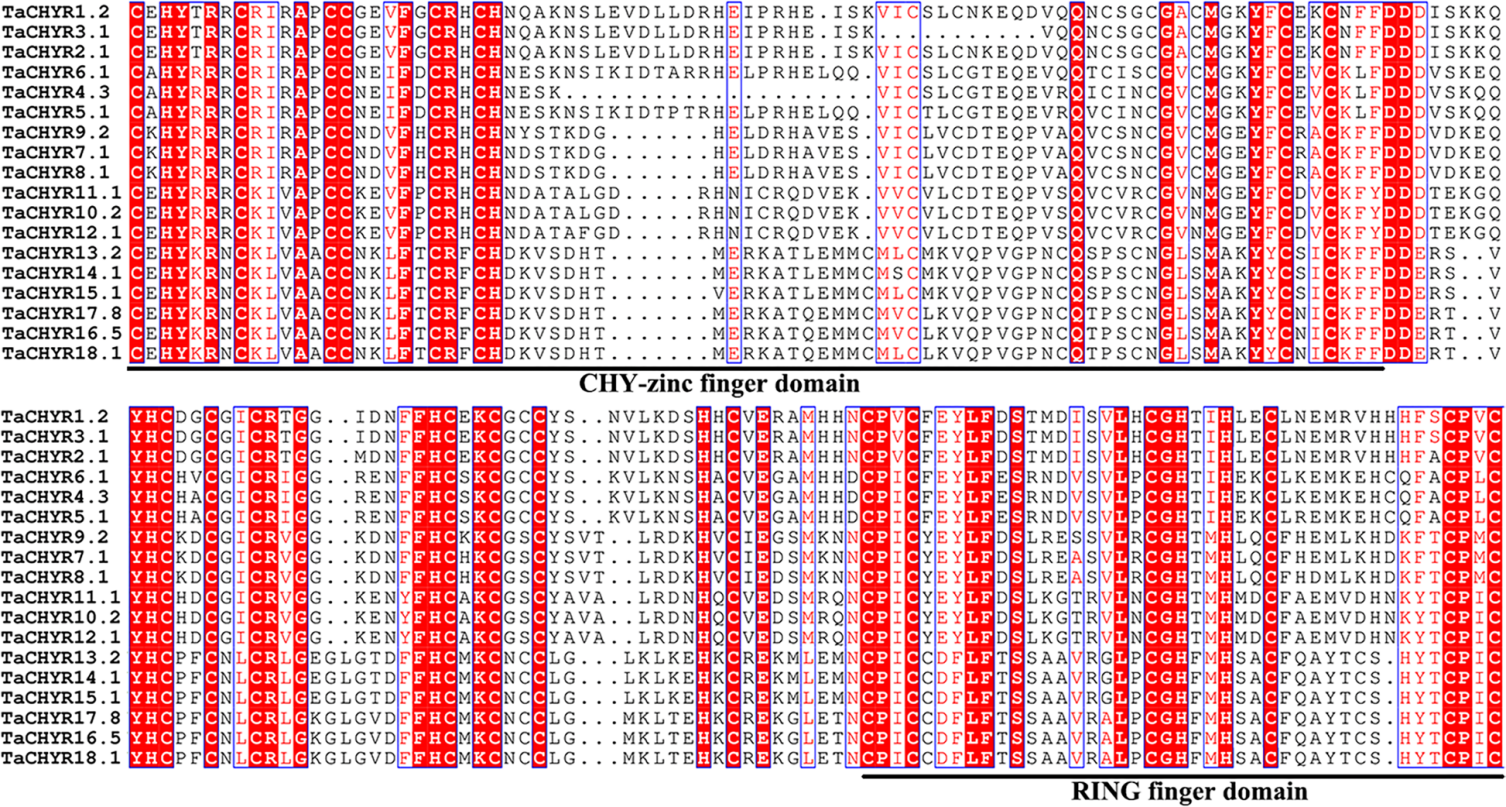
Multiple sequence alignment of the conserved domains of *TaCHYR* gene family in wheat. CHY-zinc finger and C3H2C3-type RING finger domains were underlined

To investigate the structural characteristics of *TaCHYR* genes, the exon–intron structures and conserved motifs of 18 *TaCHYR* genes were analyzed (Fig. 2). The *TaCHYR* genes contained 10–15 exons, the coding sequence (CDS) of group III members were longer than that of other two groups, and most genes with closer evolutionary relationships had similar exon–intron structures (Fig. 2B). Furthermore, the conserved domains of TaCHYRs were analyzed, the results showed that all TaCHYRs contained CHY-zinc finger, C3H2C3-type RING finger and zinc ribbon domains (Fig. 2C and Fig. 3). These results further verified the reliability of the identified *TaCHYR* gene family members. Besides, group III members contained 1–3 hemerythrin domains in the N-terminus. Meanwhile, we predicted the conserved motifs of TaCHYR gene family using MEME tools (Fig. 2C and Fig. S1). Among the detected 10 motifs, motif 2, 4 and 6 formed CHY-zinc finger domain, motif 3 and 9 made up RING finger domain. Zinc ribbon domain consisted of motif 7 and 9. Hmerythrin domain included motif 8 and 10. Additionally, motif 1, 5 and 10 were unique to group III members. These results indicated that the composition of conserved motifs were varied among different CHYR subfamilies, but TaCHYRs with closer evolutionary relationships had more similar conserved domains.

### Chromosomal location, synteny and *Ka*/*Ks* analysis of *TaCHYR*s

**Fig. 4:**
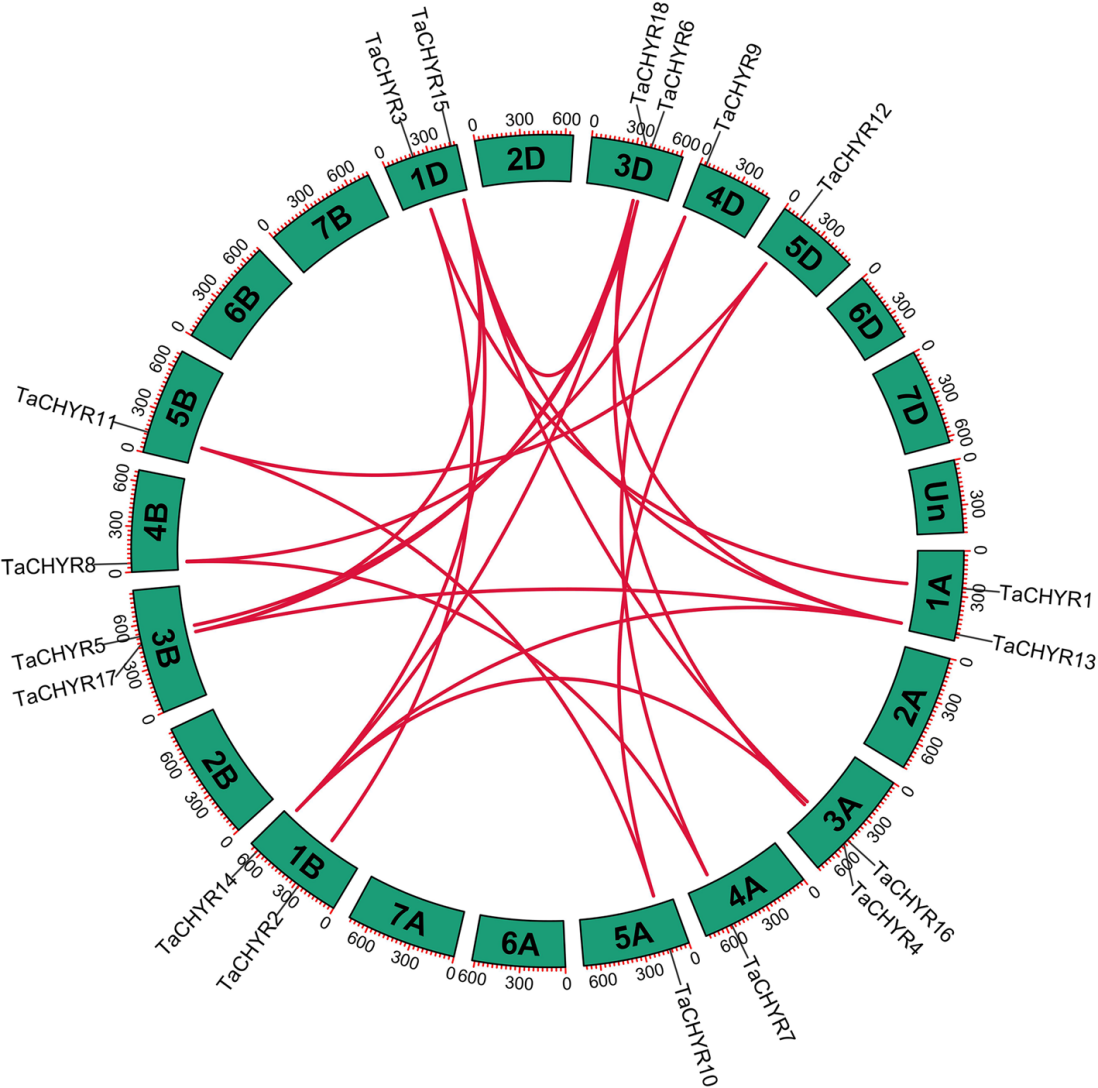
Chromosomal localizations and syntenic relationships among *TaCHYR* genes in wheat. Red lines in highlight indicated the syntenic *TaCHYR* gene pairs

The chromosomal location and synteny of *TaCHYR* gene family were analyzed according to their genomic sequences (Fig. 4 and Fig. S2). *TaCHYR* genes were distributed on chromosome 1, 3, 4, and 5. Eighteen *TaCHYR* genes were evenly distributed among A, B, and D subgenomes: *TaCHYR1*, *4*, *7*, *10*, *13* and *16* were located on A subgenome; *TaCHYR2*, *5*, *8*, *11*, *14* and *17* on B subgenome; *TaCHYR3*, *6*, *9*, *12*, *15* and *18* on D subgenome. Chromosome 1A, 1B, 1D, 3A, 3B and 3D contained two *TaCHYR* genes, respectively. Chromosome 4A, 4B, 4D, 5A, 5B and 5D contained only one *TaCHYR* genes, respectively (Fig. S2). A total of 22 paralogous *TaCHYR* gene pairs were determined in wheat genome, which all undergone WGD (whole genome duplications) or segmental duplication events (Fig. 4 and Table S3). Eighteen genes were all involved in gene duplication events, and these results indicated that *TaCHYR* genes might be produced by segmental duplication events and these duplication events might contribute to the evolution of *TaCHYR* genes. Moreover, the non-synonymous (*Ka*) and synonymous substitution (*Ks*) ratios were calculated to investigate selective pressure of these 22 paralogous gene pairs (Table S3). The *Ka*/*Ks* ratios of paralogous gene pairs were all less than 1, indicating these *TaCHYR* genes were under strong purifying selection to maintain the function of *TaCHYR* gene family.

**Fig. 5:**
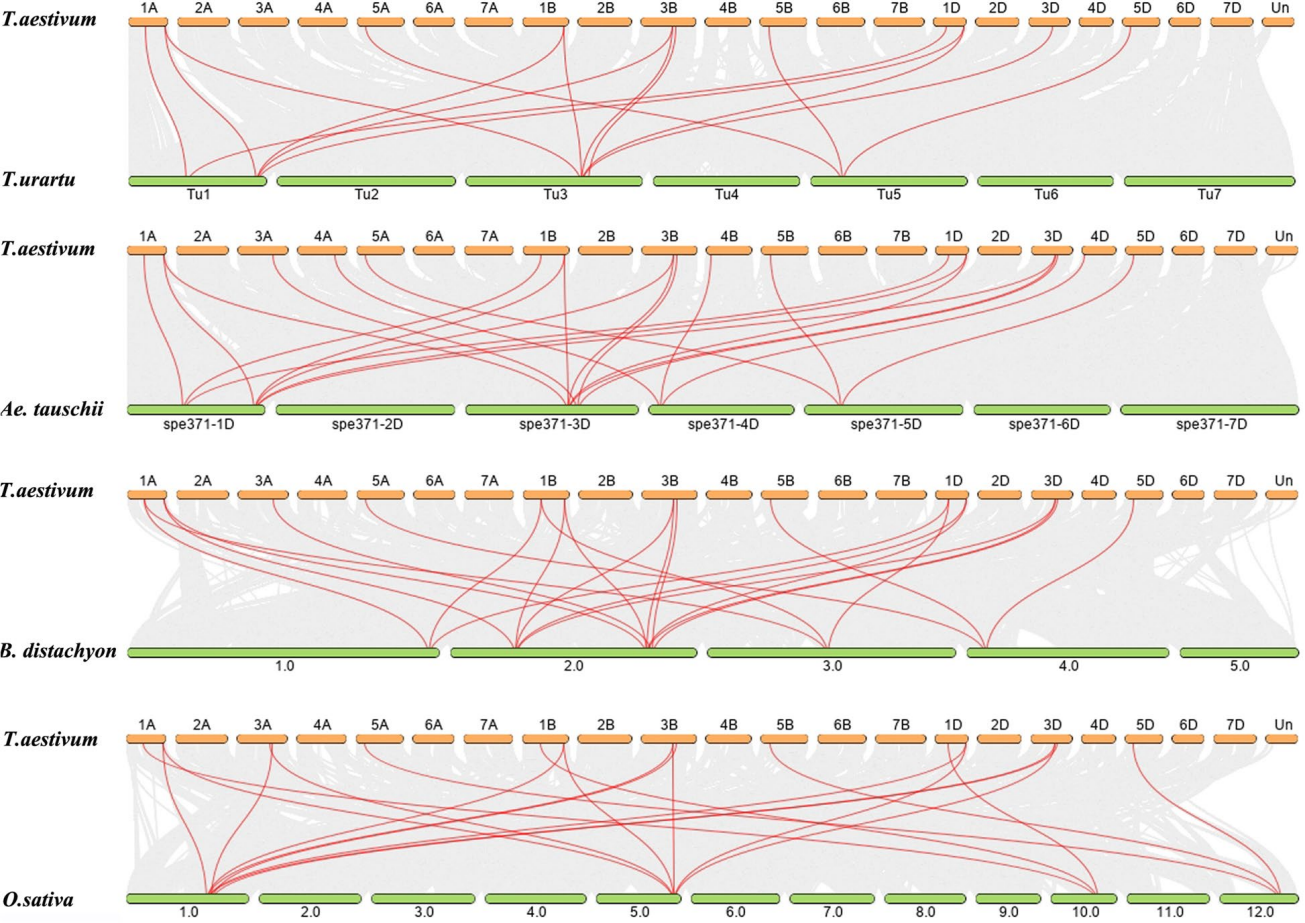
Syntenic relationships between *TaCHYR* genes in *T. aestivum* with other *CHYR* genes in other four representative plant species. Gray lines in the background indicated the collinear blocks within *T. aestivum* and other plant genomes. Red lines in highlight indicated the syntenic *CHYR* gene pairs

Furthermore, we analyzed the synteny of *CHYR* genes between *T. aestivum* (AABBDD, hexaploid) with *T. urartu* (AA, diploid), *Ae. tauschii* (DD, diploid), *B. distachyon* (diploid) and *O. sativa* (diploid) (Fig. 5 and Table S4). The orthologous gene pairs of *CHYR* genes were identified between *T. aestivum* with *T. urartu* (15), *Ae. tauschii* (22), *B. distachyon* (22) and *O. sativa* (20), respectively. And 11, 17, 14 and 15 *TaCHYR* genes showed collinearity relationships with *CHYR* genes in *T. urartu*, *Ae. tauschii*, *B. distachyon* and *O. sativa*, respectively. Moreover, the synteny analysis showed 3 orthologous gene pairs which located on the same chromosomes between wheat A subgenome and *T. urartu* were identified with two on chromosome 1A (*TaCHYR1*/*TuG1812G0100001751*, *TaCHYR13*/*TuG1812G0100004139*), and one on chromosome 5A (*TaCHYR10*/*TuG1812G0500001083*). Similarly, 6 orthologous gene pairs between wheat D subgenome and *Ae. tauschii* were identified which located on the same chromosomes with two on chromosome 1D (*TaCHYR3*/*AET1Gv20368300*, *TaCHYR15*/*AET1Gv20902300*), two on chromosome 3D (*TaCHYR6*/*AET3Gv20682500*, *TaCHYR18*/*AET3Gv20635000*), one on chromosome 4D (*TaCHYR9*/*AET4Gv20135500*), and one on chromosome 5D (*TaCHYR12*/*AET5Gv20264800*) (Fig. 5 and Table S4). These *TaCHYR* genes might be originated from orthologous genes in *T. urartu* and *Ae. tauschii* with the occurrence of natural hybridization events.

### *Cis*-elements analysis of *TaCHYR* genes

**Fig. 6:**
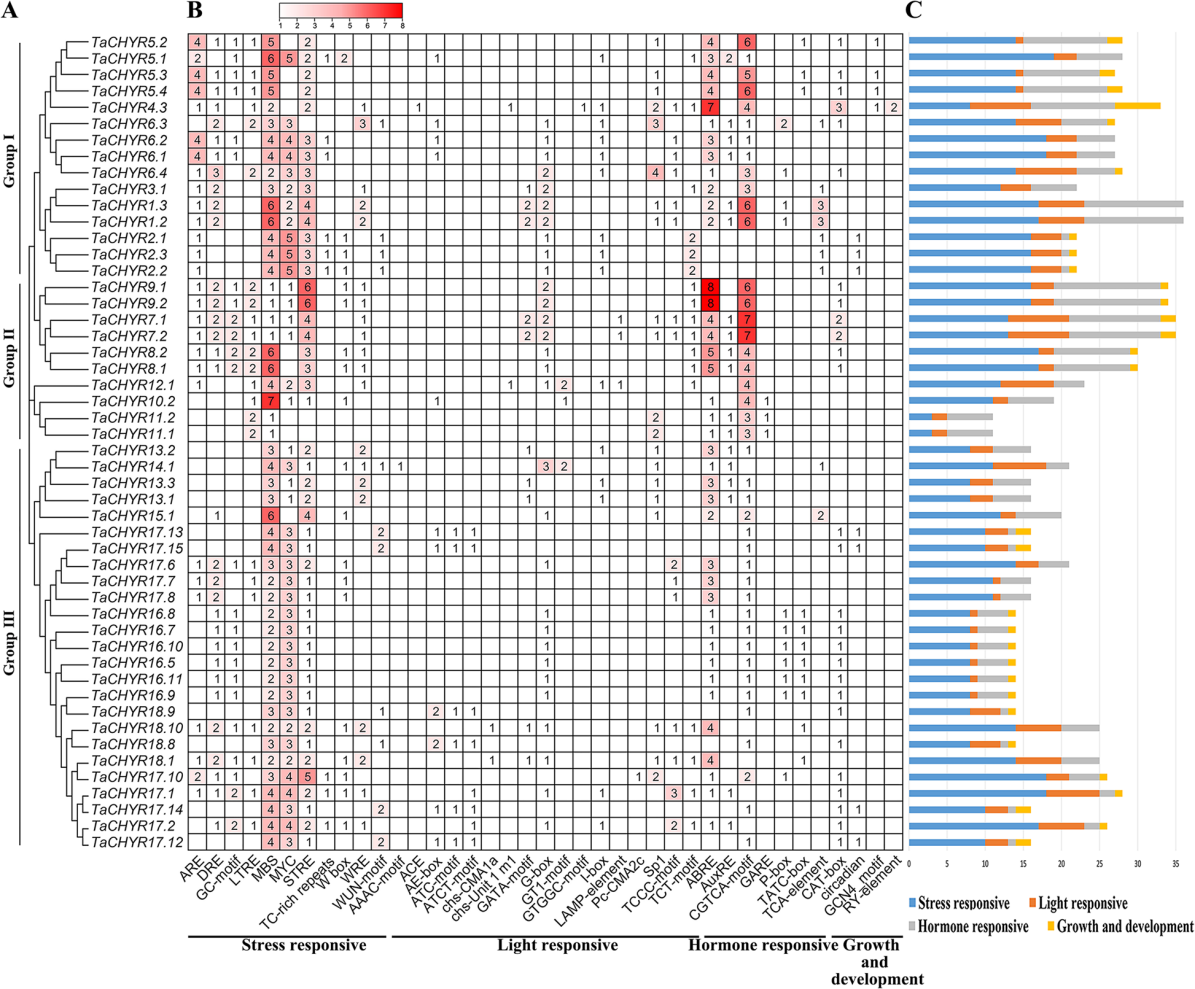
Analysis of *cis*-elements in *TaCHYR* promoters. **A** Phylogenetic analysis of *TaCHYR* gene family. **B** *Cis*-elements in the promoters of *TaCHYR* genes. The different colors and numbers of grids indicated the numbers of different promoter elements. **C** The histograms of different colors represented the sum of the *cis*-elements in each category

The variable *cis*-elements in the gene promoters might indicate that these genes perform different functions in plant growth, development and various stress responses. To further investigate the functions of *TaCHYR* genes, 1.5 kb of the upstream region of each *TaCHYR* gene transcription start site (TSS) was extracted and then analyzed by using the PlantCARE database to identify and count the *cis*-elements (Fig. 6). These *cis*-elements were divided into four categories, i.e., stress responsive-, light responsive-, hormone responsive-, and growth and development related-elements. The promoter of *TaCHYR* genes included masses of stress and hormone responsive-elements, especially DRE (drought-responsive element), MBS (MYB binding site), MYC and STRE (stress-responsive element) among stress responsive-elements, and ABRE (ABA-responsive element) and CGTCA-motif (MeJA-responsive element) among hormone responsive-elements. Additionally, group I members had abundant ARE (anaerobic induction-responsive element). G-box abounded in *TaCHYR* promoters, which could interact with bZIP or bHLH transcription factors to participate in biological processes [19]. These results indicated that the *TaCHYR* genes might play significant roles in plant growth, development and respond to various stresses in wheat.

### GO annotation and network construction of *TaCHYR*s

**Fig. 7:**
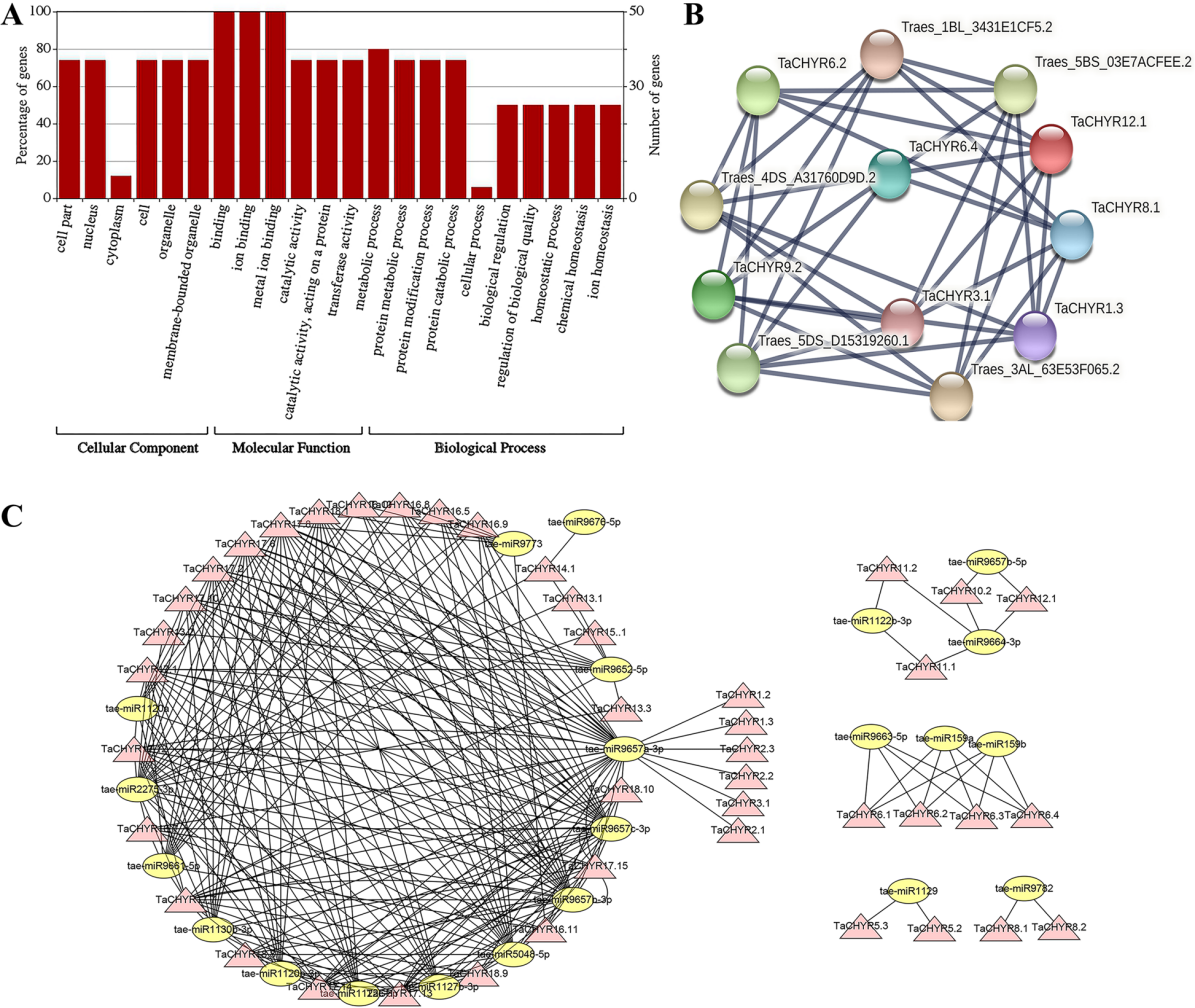
Gene ontology (GO) annotation **A** and protein–protein interactions **B** and miRNA targeting **C** analysis of *TaCHYR* genes. **A** The GO terms were showed on the X axis, and the number of genes were showed on the Y axis. **C** Pink and yellow boxes indicated *TaCHYR* genes and miRNAs in wheat, respectively

To further understand the function of *TaCHYR* from molecular levels, all TaCHYR proteins were annotated by gene ontology (GO) (Fig. 7A and Table S6). These TaCHYR proteins were assigned with 22 GO terms belonging to the cellular component, molecular function and biological process (Fig. 7A). Under the cellular component category, most TaCHYR proteins (74%) were located in the nucleus (GO:0005634). Under the molecular function category, all TaCHYRs were involved in metal ion binding (GO:0046872). Under the biological process category, most TaCHYRs were involved in protein metabolic process (GO:0019538), protein modification process (GO:0036211), protein catabolic process (GO:0030163), and homeostatic process (GO:0042592). According to protein–protein interactions (PPIs) analysis, TaCHYR proteins could bind other TaCHYR family members or other E3 ubiquitin-protein ligases to form heterodimers (Fig. 7B and Table S7).

The putative microRNA (miRNA) targeting *TaCHYR* genes were analyzed using psRNATarget tools [20], the results demonstrated that 21 miRNAs were identified to target 15 *TaCHYR* genes encoding 43 transcripts, while 3 *TaCHYR* genes were not targeted by miRNAs (Fig. 7C and Table S8). The most transcripts were degraded via cleavage (75.68%), while the rest of *TaCHYR* transcripts were inhibited by miRNA via translation (24.32%). The tae-miR9657a-3p, tae-miR9657b-3p, and tae-miR9652-5p targeted 6 (*TaCHYR1*, *2*, *3*, *16*, *17* and *18*), 5 (*TaCHYR10*, 1*2*, *16*, *17* and *18*) and 4 (*TaCHYR13*, *14*, *15* and *17*) *TaCHYR* genes, respectively. Besides, tae-miR2275-3p, tae-miR5048-5p, tae-miR9657c-3p, tae-miR9661-5p, tae-miR9664-3p and tae-miR9773 targeted 3 *TaCHYR* genes, respectively. These results provided a valuable foundation for future functional investigations of *TaCHYR* genes.

### Expression patterns of *TaCHYR* Genes in different tissues

**Fig. 8:**
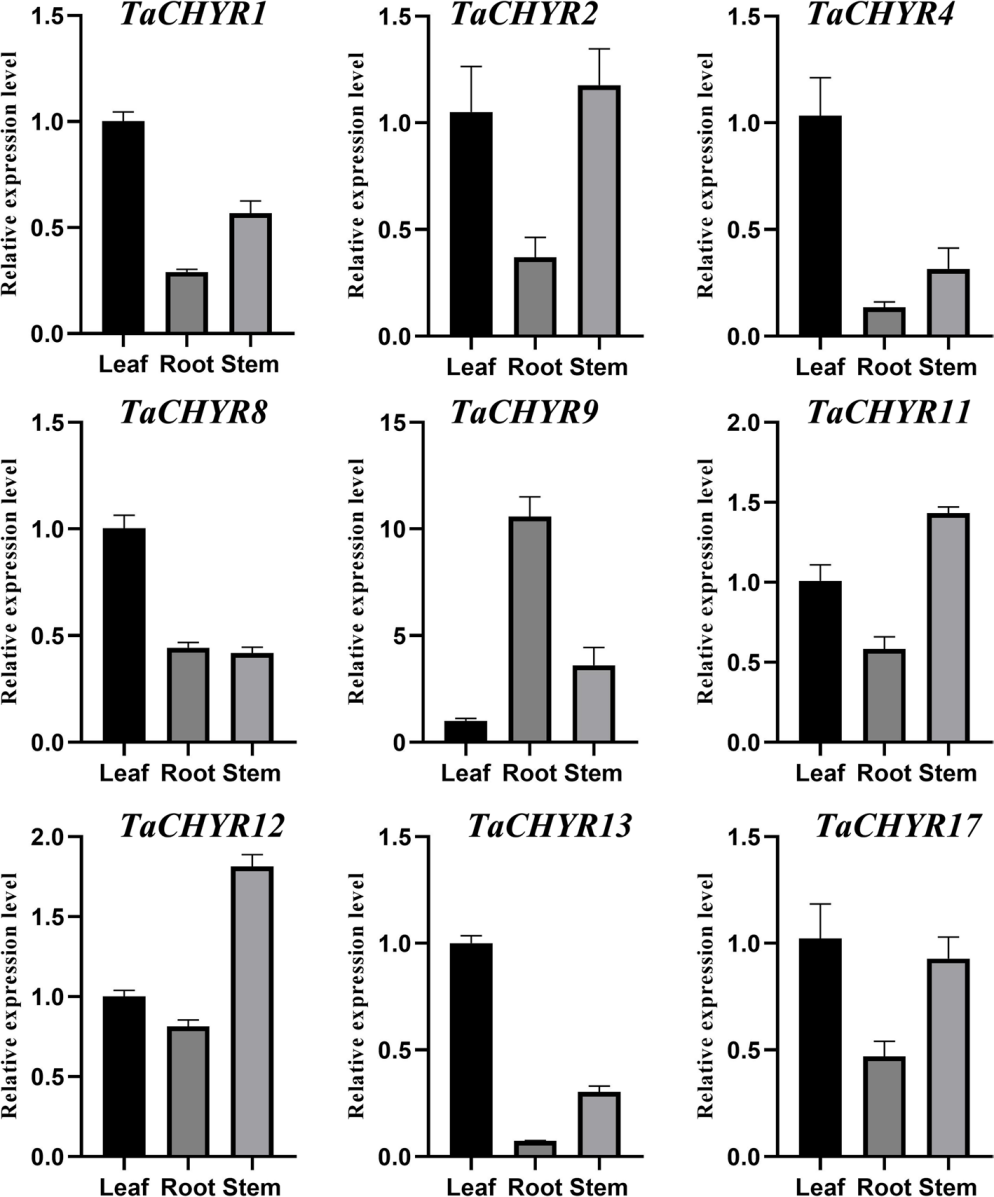
Expression levels of *TaCHYR* genes in the roots, stems and leaves during wheat seedlings stage. The expression level of the wheat *actin* gene was used as the internal control to standardize the RNA samples for each reaction. The values were the mean ± SE from three samples

To investigate the tissue-specific expression patterns of the *TaCHYR* genes, the gene expression levels of 9 selected *TaCHYR* genes belonging to group I (*TaCHYR1*, *2* and *4*), group II (*TaCHYR8*, *9*, *11* and *12*) and group III (*TaCHYR13* and *17*) members were determined by using real-time PCR in roots, stems, and leaves tissues during wheat seedling stage (Fig. 8). Most of selected 9 *TaCHYR* genes exhibited higher expression levels in leaves, such as *TaCHYR1*, *4*, *8*, *13*, and *17* were highly expressed in leaves. Group I (*TaCHYR1*, *2* and *4*) and group III (*TaCHYR13* and *17*) members were predominantly expressed in leaves and stems, followed by roots. Group II (*TaCHYR8*, *9*, *11* and *12*) members showed differential expression profiles in roots, stems, and leaves tissues. *TaCHYR8* and *TaCHYR9* showed highest expression levels in leaves and roots, respectively. *TaCHYR11* and *TaCHYR12* exhibited the highest expression levels in stems, followed by leaves, and finally in roots. In particular, most paralogous genes showed similar expression patterns, e.g., *TaCHYR1*/*2*, *TaCHYR11*/*12*, and *TaCHYR13*/*17*. However, the paralogous genes *TaCHYR8*/*9* had different expression profiles in various tissues, indicating *TaCHYR8* and *9* might have diverse function due to ecological or environmental adaptation. These might be caused by modification in their promoters, some special regulatory elements, or functional segregation with a long-term evolutionary process.

### Expression patterns of *TaCHYR* genes under abiotic stress

**Fig. 9:**
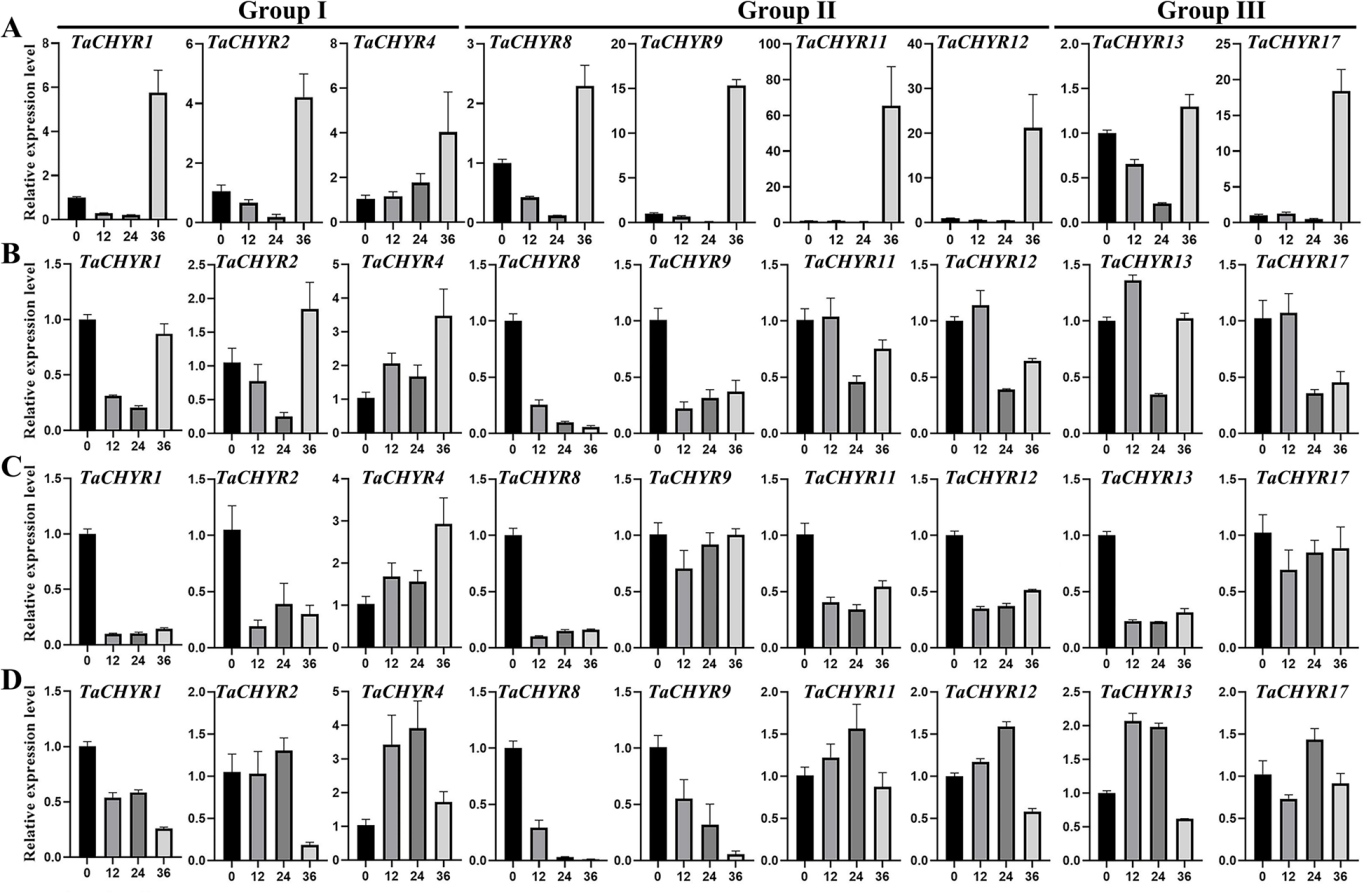
Expression patterns of *TaCHYR* genes in response to PEG (polyethylene glycol) **A**, salt **B**, heat **C** and cold **D** treatments determined by real-time PCR. Wheat seedling leaves were sampled after 0, 12, 24 and 36 h under stress conditions. The expression level of the wheat *actin* gene was used as the internal control to standardize the RNA samples for each reaction. The values were the mean ± SE from three samples

To insight into the function of *TaCHYR* genes under abiotic stress, real-time PCR was used to determine the expression patterns of these 9 *TaCHYR* genes under PEG, salt and heat and cold stress in leaves during the wheat seedling stage (Fig. 9). Under PEG stress, all these *TaCHYR* genes were up-regulated and reached the highest expression level at 36 h. *TaCHYR11* and *TaCHYR12* were significantly up-regulated more than 65-fold and 21-fold under PEG stress compared with the control, respectively (Fig. 9A). These *TaCHYR*s exhibited differential expression patterns in seedling leaves in response to salt, cold and heat stress. In salt stress condition, *TaCHYR2* and *4* were obviously up-regulated and reached the highest expression level at 36 h, and *TaCHYR8* and *9* were significantly down-regulated (Fig. 9B). After heat stress treatment, most these *TaCHYR* genes were down-regulated compared with control, except *TaCHYR4* was up-regulated (Fig. 9C). Under cold stress, *TaCHYR2*, *4*, *11*, *12*, *13* and *17* were obviously up-regulated, and the expression of *TaCHYR1*, *8* and *9* were significantly suppressed compared with control (Fig. 9D). Moreover, the paralogous genes had similar expression patterns, e.g., *TaCHYR11/12* had almost consistent expression patterns under PEG, salt, heat or cold stress. These results suggested that *TaCHYR* genes had different response to different stress and play various regulatory roles in abiotic stress resistance.

### Negative regulation of dehydration and salt stress tolerance in recombinant yeast cells

**Fig. 10:**
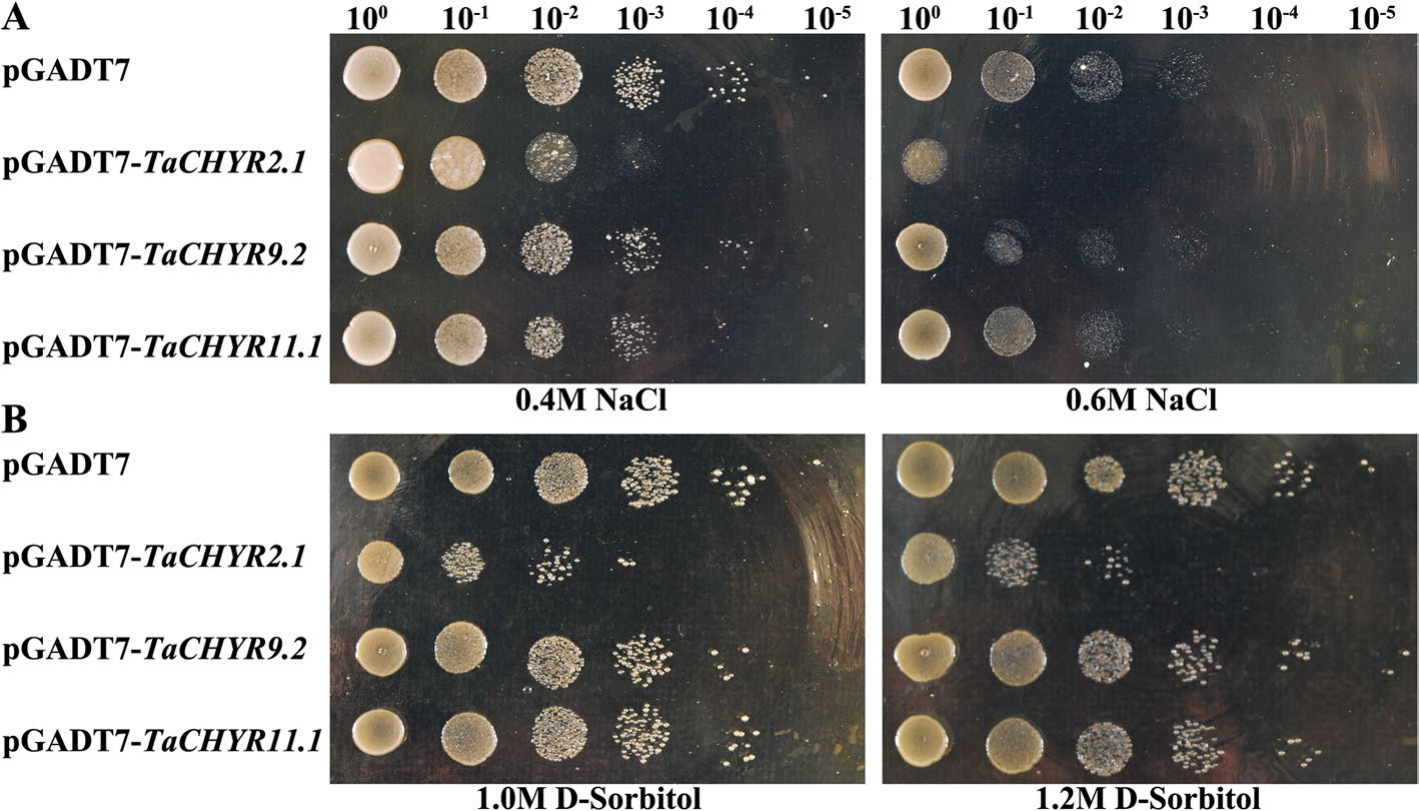
The ability of the tolerance in response to salt **A** and dehydration **B** stress in recombinant yeast cells. The osmotic-sensitive yeast mutants Δ*hog1* carrying the empty vector (pGADT7, negative control), pGADT7-*TaCHYR2.1*, pGADT7-*TaCHYR9.2*, or pGADT7-*TaCHYR11.1* were spotted onto YPD medium plates containing NaCl (0.4 and 0.6 M) or D-Sorbitol (1.0 and 1.2 M), and cultured at 30℃ for 3 d

According to the expression profiles, three *TaCHYR* genes (*TaCHYR2.1*, *TaCHYR9.2*, and *TaCHYR11.1*) were selected to clone into pGADT7 vector, and then transformed into the osmotic-sensitive yeast mutant BY4741 (Δ*hog1*) to verify the ability in response to stress tolerance in yeast mutant cells (Fig. 10). The results suggested that the growth of the Δ*hog1* yeast cells containing these recombinant vectors (pGADT7-*TaCHYR2.1*, pGADT7-*TaCHYR9.2* or pGADT7-*TaCHYR11.1*) were inhibited in YPD medium containing NaCl (0.4 and 0.6 M) and D-Sorbitol (1.0 and 1.2 M) compared with the control (pGADT7 empty vector), and the growth of yeast cells were inhibited more obviously with the increase of NaCl and D-Sorbitol concentration (Fig. 10A and 10B). Moreover, the growth of yeast expressing *TaCHYR2.1* was severely inhibited in salt and dehydration treatment conditions. The growth of yeast expressing *TaCHYR9.2* was similar to *TaCHYR11.1*, which was slightly inhibited under salt and dehydration stress. These results revealed that *TaCHYR2.1*, *TaCHYR9.2* and *TaCHYR11.1* might participate in protein catabolic and homeostatic process under salt and dehydration stress in wheat.

## Discussion

Previously, *CHYR* genes have been identified in diverse plants, such as maize, *Arabidopsis*, rice and soybean [2, 8, 14, 18]. However, *CHYR* genes have not been genome-wide identified in wheat. In this study, 18 *CHYR* genes were identified in wheat and classified into 3 groups (Fig. 1). All TaCHYR proteins contained CHY-zinc finger, C3H2C3-type RING finger and zinc ribbon domains (Fig. 2C). Besides, there were 1–3 hemerythrin domains in the N-terminus of group III members, which may play vital roles in regulating iron homeostasis [8].

Previous studies showed that *CHYR* genes occurred during evolution of green plants, group I members were present in Bryophyta, Pteridophyta and Gymnosperms, while group II members were absent, suggesting the differentiation of *CHYR*s between group I and II members might occur after angiosperm differentiation [2]. Therefore, group I and II members could be found to coexist in monocotyledonous (wheat and rice) and dicotyledonous (*Arabidopsis* and soybean) plants (Fig. 1 and Fig. 5). Group III members were far away from the other two groups in plants evolution process, indicating that they might originated from different ancestors [2]. In this study, we also analyzed the synteny relationships of *CHYR* genes between the wheat and other plant species, three orthologous gene pairs that located on the same chromosomes between wheat A subgenome and *T. urartu* were identified. Similarly, six orthologous gene pairs which located on the same chromosomes between wheat D subgenome and *Ae. tauschii* were identified (Fig. 5 and Table S4). These *TaCHYR* genes might be originated from orthologous genes in *T. urartu* and *Ae. tauschii* with the occurrence of natural hybridization events. Moreover, 22 paralogous gene pairs were identified among 18 *TaCHYR* genes in wheat genome, which all undergone WGD or segmental duplication events and a strong purifying selection pressure (Fig. 4 and Table S3). These results indicated that WGD or segmental duplications played crucial roles in the expansion of the *TaCHYR* genes.

*CHYR* genes participated in various biological processes to play vital roles in plant growth, development and various stress responses [3]. Real-time PCR results indicated that most of selected 9 *TaCHYR* genes exhibited higher expression levels in leaves during wheat seedling stage (Fig. 8), suggesting these *TaCHYR*s might play an important function in leaves. The tissue-specific expression of *TaCHYR* genes mainly related with the variable *cis*-elements present in its promoters (Fig. 6). Under PEG stress, all *TaCHYR* genes were up-regulated, which might be regulated by masses of stress and hormone responsive-elements in the *TaCHYR* promoter regions (Fig. 6 and Fig. 9A). Moreover, the similar expression patterns were detected in the paralogous genes, e.g., *TaCHYR11*/*12* were significantly induced more than 65-fold and 21-fold compared with the control after PEG treatment, respectively. However, less stress and hormone responsive-elements were identified in the promoter of *TaCHYR11*, probably due to the lack of the 425 bp genomic sequences in *TaCHYR11* promoter region (Fig. 6 and Fig. S3). Therefore, we speculated that *TaCHYR* genes played vital roles when plants suffered dehydration stress. MiRNAs also played an important role in regulating the expression of downstream genes [21]. Here, 21 miRNAs were identified, and tae-miR9657a-3p targeted 6 *TaCHYR* genes, implying tae-miR9657a-3p played a key role in regulating the expression of *TaCHYR* genes (Fig. 7C and Table S8).

GO annotation analysis indicated *TaCHYR*s were E3 ubiquitin-protein ligases (Fig. 7A and Table S6), and regulated the plant adaptive response to abiotic stress via ABA-mediated signaling, and especially through modulating the stability of bZIP and bHLH transcription factor [19, 22]. Previous studies have showed that *CHYR1* promoted ABA-induced stomatal closure, reactive oxygen species production, and plant drought tolerance in *Arabidopsis*, the similar results were also found in poplar [3, 13]. CHYR gene *CaASRF1* positively modulates ABA signaling and the drought stress response via modulation of bZIP transcription factor CaAIBZ1 stability [22]. In contrary, the rice E3 ubiquitin-protein ligase drought-induced SINA protein 1 (OsDIS1) negatively regulated drought stress response through transcriptional or post-translational regulation of various stress-related genes [23]. Rice *OsRZFP34* gene enhances stomatal opening, leaf cooling and ABA insensitivity [14]. *Arabidopsis* E3 ubiquitin ligase PUB11 negatively regulated drought tolerance by degrading the receptor-like protein kinases LRR1 and KIN7 [24]. The C3HC4-type RING finger E3 ubiquitin ligase TaSADR1 negatively regulates drought resistance in transgenic *Arabidopsis* [25]. In our study, the growth of yeast cells expressing *TaCHYR2.1*, *TaCHYR9.2* and *TaCHYR11.1* were inhibited in salt and dehydration treatment conditions, probably caused by the ubiquitination of interacting proteins resulting in degradation of stress-related proteins. In the future, these genes would be knocked out through CRISPR technology to obtain drought-resistant wheat. These results still require to be confirmed in wheat, and E3 ubiquitin-protein ligase activity of TaCHYRs also require further experimental verification.

## Conclusions

In this study, 18 *TaCHYR* genes were identified, which can be divided into three groups. All *TaCHYR* genes contained CHY-zinc finger, C3H2C3-type RING finger and zinc ribbon domains, and group III members included 1–3 hemerythrin domains. *TaCHYR* genes were distributed on chromosome 1, 3, 4, and 5, and evenly distributed among A, B, and D subgenomes. *Ka*/*Ks* analysis showed that the *TaCHYR*s undergone a strong purifying selection pressure during the evolution process. Twenty-two paralogous gene pairs were identified in wheat, and 15, 22, 22 and 20 orthologous gene pairs were identified between wheat with *T. urartu*, *Ae. tauschii*, *B. distachyon* and *O. sativa*, respectively. The promoters of *TaCHYR* genes contained masses of stress and hormone responsive-elements. Real-time PCR results suggested that most of selected 9 *TaCHYR* genes exhibited higher expression levels in leaves during wheat seedling stage. All these *TaCHYR* genes were up-regulated after PEG treatment, and these *TaCHYR*s exhibited differential expression profiles in response to salt, cold and heat stress. The growth of yeast cells overexpressing *TaCHYR2.1*, *TaCHYR9.2* and *TaCHYR11.1* was suppressed under salt and dehydration stress. Moreover, gene ontology (GO) annotation, protein interaction and miRNA regulatory network of *TaCHYR* genes were analyzed. These results provide useful information for further functional studies of *TaCHYR* genes, and lay a foundation to improve wheat quality traits in molecular breeding under abiotic stress.

## Methods

### Identification of the *CHYR* family genes

The genome sequence of *T. aestivum* was downloaded from EnsemblPlants database (http://plants.ensembl.org/index.html). The Hidden Markov Model (HMM) profiles (http://pfam.xfam.org) of CHY zinc-finger domain (PF05495) obtained from Pfam database (http://pfam.xfam.org) were used to HMM search against the local genome database of *T. aestivum* using TBtools [26]. All the identified TaCHYR candidates were verified by using Pfam (http://www.ebi.ac.uk/Tools/hmmer/) and SMART database (http://smart.embl.de/), then we retrieved 18 *TaCHYR* genes. The physiological and biochemical parameters of the TaCHYR proteins were analyzed by WheatOmics 1.0 (http://202.194.139.32/) [27], and the subcellular localization of the TaCHYR proteins were predicted using Plant-mPLoc (http://www.csbio.sjtu.edu.cn/bioinf/plant-multi/).

### Phylogenetic relationships, gene structures and domains analysis

The phylogenetic tree was constructed by the neighbor-joining (NJ) method with 1000 bootstrap replicates using MEGA11 software and Evolview online service [28, 29]. The amino acid sequences of AtCHYRs and GmCHYRs were obtained from a previous report [28, 29]. The exon–intron structures were identified by comparing CDS and genomic DNA sequences using TBtools [26]. The conserved domains and motifs were annotated using the SMART database (http://smart.embl.de/) and MEME online server (http://meme-suite.org/index.html).

### Chromosomal location, synteny and *Ka*/*Ks* analysis

The position of *TaCHYR* genes on chromosomal were obtained according to wheat genome annotation data and then marked on the chromosomes by using the TBtools and circos [26, 30]. Multiple collinear scanning toolkits (MCScanX) were used to detect the gene replication events [31]. TBtools was used to determine the *Ka* (non-synonymous rate), *Ks* (synonymous rate), and *Ka*/*Ks* ratios of the syntenic gene pair with the Nei-Gojobori (NG) method [26].

### GO annotation, microRNA targeting and protein interactions analysis

Gene Ontology (GO) annotation of TaCHYR proteins was analyzed using the eggNOG-mapper (http://eggnog-mapper.embl.de/) and OmicsBox (https://www.biobam.com/), then displayed by the WEGO2.0 website (https://wego.genomics.cn/). Protein–protein interactions (PPIs) were predicted using the STRING database (https://string-db.org/). The combined score > 0.9 in the STRING database was used to confirm the interaction network. The microRNA (miRNA) targeting *TaCHYR* genes was searched using the psRNATarget tools (http://plantgrnbb.noble.org/psRNATarget) with default parameters [20]. The Cytoscape software (https://cytoscape.org/) was used to visualize the regulatory network [32].

### *Cis*-element analysis in the promoter

The promoter sequences, 1.5 kb upstream sequences of the transcription start site (TSS) of the *TaCHYR* genes, were acquired from the wheat database, and the *cis-*elements in the promoters were analyzed using PlantCARE database (http://bioinformatics.psb.ugent.be/webtools/plantcare/html/) [33].

### Real-time PCR analysis

Wheat seeds of “Chinese Spring”, which were obtained from the Northwest A&F University, were germinated on moist filter paper at 25/18 ℃ (day/night) with a photoperiod of 16 h light/8 h dark at Henan University of Science and Technology on 20 December 2021. For abiotic stress treatment, seedlings grown in hydroponic culture for two weeks were exposed to 20% PEG6000 (w/v), high salinity (300 mM NaCl), high temperature (42 ℃), and cold (4 ℃). In each treatment, the leaf tissues were collected every 12 h for 36 h, frozen in liquid nitrogen, and stored at − 80 ℃ [34].

RNAiso Plus (Takara) was used to isolate total RNA from each frozen sample, and the first-strand cDNA was synthesized from total RNA (1 μg) by using Prescript III RT ProMix (CISTRO) according to the manufacturer’s instructions. The sequence was amplified using gene-specific primers (Table S9) with 2 × Ultra SYBR Green qPCR Mix (CISTRO), and the *actin* gene was used as an internal control. The real-time PCR cycling parameters were 95 °C for 30 s, followed by 45 cycles at 95 °C for 5 s and 60 °C for 30 s, with a melting curve analysis. All reactions were performed in triplicate to ensure the reproducibility of the results.

## Stress tolerance assay in yeast cells

The coding sequence (CDS) of *TaCHYR* genes were cloned into a pGADT7 vector using the BM seamless cloning kit (Biomed), and then transformed into osmotic-sensitive yeast mutants Δ*hog1* (*MATa*, *his3∆1*, *leu2∆0*, *met15∆0*, *ura3∆0*, *hog1*::*KanMX4*). The primers were shown in Table S9. To analyze stress resistance, the yeast cells carrying the empty vector (pGADT7), pGADT7-*TaCHYR2.1*, pGADT7-*TaCHYR9.2*, or pGADT7-*TaCHYR11.1* were cultured in YPD liquid medium (1% yeast extract, 2% peptone, 2% glucose) at 30℃ until density reached an OD_600_ of 1.0, then serially diluted (10^0^, 10^–1^, 10^–2^, 10^–3^, 10^–4^, 10^–5^) with ddH_2_O. The cells were spotted onto YPD medium plates (1% yeast extract, 2% peptone, 2% glucose, 2% agar) containing NaCl (0.4 and 0.6 M) or D-Sorbitol (1.0 and 1.2 M), and cultured at 30℃ for 3 d.

## Supplementary Information

**Additional file 1:** **Fig. S1.** Conserved motifs of TaCHYR proteins in wheat

**Additional file 2: ** **Fig. S2.** Chromosomal localizations of *TaCHYR* genes in wheat. Group I, II and III members were indicated by red, black and blue, respectively.

**Additional file 3:** **Fig. S3.** The promoter sequences of *TaCHYR11*. The transcription start site (TSS) and start codon were indicated by red and yellow, respectively.

**Additional file 4:** **Table S1.** The characteristics of *CHYR* genes in wheat

**Additional file 5: Table S2. ***CHYR* genes used in the phylogenetic tree construction 

**Additional file 6: Table S3. **Paralogous* CHYR *gene pairs among *T.aestivum* 

**Additional file 7:** **Table S4.** Orthologous relationships between *TaCHYR* genes in* T. aestivum* with other *CHYR* genes in *T. urartu*, *Ae.tauschii*, *B. distachyon*, and *O. sativa*

**Additional file 8:** **Table S5.** The* cis*-elements analysis in the promoter regions of *TaCHYR* genes

**Additional file 9:** **Table S6.** Gene ontology (GO) annotation of *TaCHYR* genes in *T. aestivum*

**Additional file 10: Table S7. **The protein-protein interaction network between TaCHYRs and other proteins in wheat

**Additional file 11:** **Table S8.** The regulatory network between the putative miRNAs and their targeted wheat *CHYR* genes

**Additional file 12:** **Table S9. **Specific primers used in the study

## Data Availability

Wheat seeds of “Chinese Spring”, which were obtained from the Northwest A&F University, were germinated on moist filter paper at 25/18 ℃ (day/night) with a photoperiod of 16 h light/8 h dark at Henan University of Science and Technology on 20 December 2021. All of the datasets supporting the results of this article are included within the article and its Additional files. The DNA and Protein sequences of *TaCHYR* are available in the EnsemblPlants database (http://plants.ensembl.org/index.html), and the gene ID provided in Additional file 3 Table S1. The amino acid sequences of AtCHYR and GmCHYR were obtained from The Arabidopsis Information Resource (https://www.arabidopsis.org/) and Phytozome database (https://phytozome-next.jgi.doe.gov/pz/portal.html), respectively, and the gene ID provided in Additional file 4 Table S2. The genomes of *T. aestivum*, *T. urartu*, *Ae. tauschii*, *B. distachyon* and *O. sativa* were obtained from the EnsemblPlants database (http://plants.ensembl.org/index.html).

## Abbreviations

CHYR: CHY zinc-finger and RING finger protein
CDS: Coding sequence
BTS/BTSL: BRUTUS/BRUTUS-like
HRZ: Hemerythrin RING zinc-finger
HR: Hypersensitive responses
WGD: Whole genome duplications
bHLH: Basic helix-loop-helix
DRE: Drought-responsive element
MBS: MYB binding site
STRE: Stress-responsive element
ABRE: ABA-responsive element
CGTCA-motif: MeJA-responsive element
GO: Gene ontology
PPI: Protein-protein interaction
ABA: Abscisic acid
