# Supplementary figures and images for: Genome-wide analysis and functional characterization of CHYR gene family associated with abiotic stress tolerance in bread wheat (Triticum aestivum L.)
Source: BMC Plant Biol. 2022 Apr 20;22:204. doi: 10.1186/s12870-022-03589-7 (PMC9019960; doi:10.1186/s12870-022-03589-7)

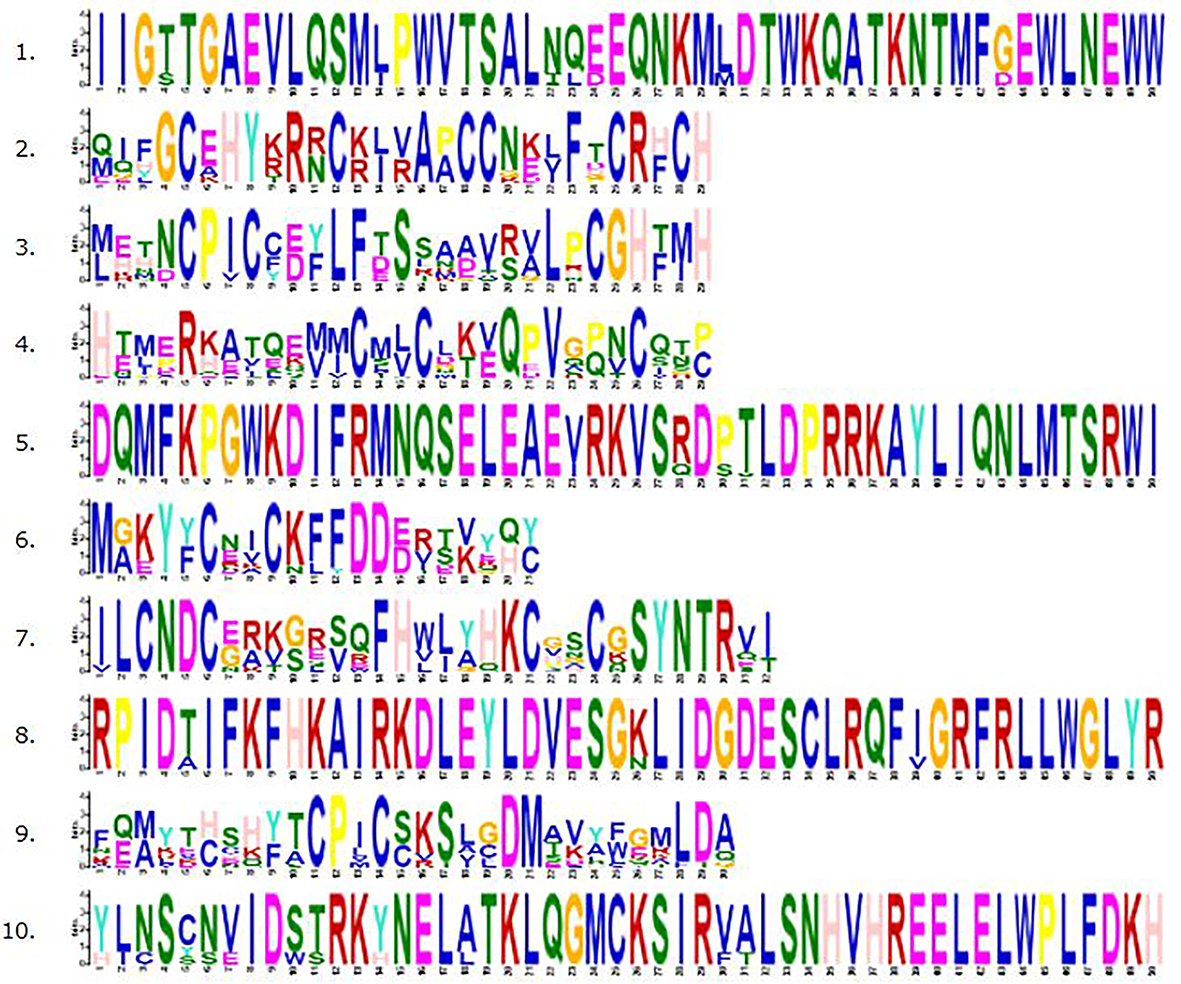

Supplement: Supplementary file 1 — Additional file 1: Fig. S1. Conserved motifs of TaCHYR proteins in wheat [file 12870_2022_3589_MOESM1_ESM.tif]

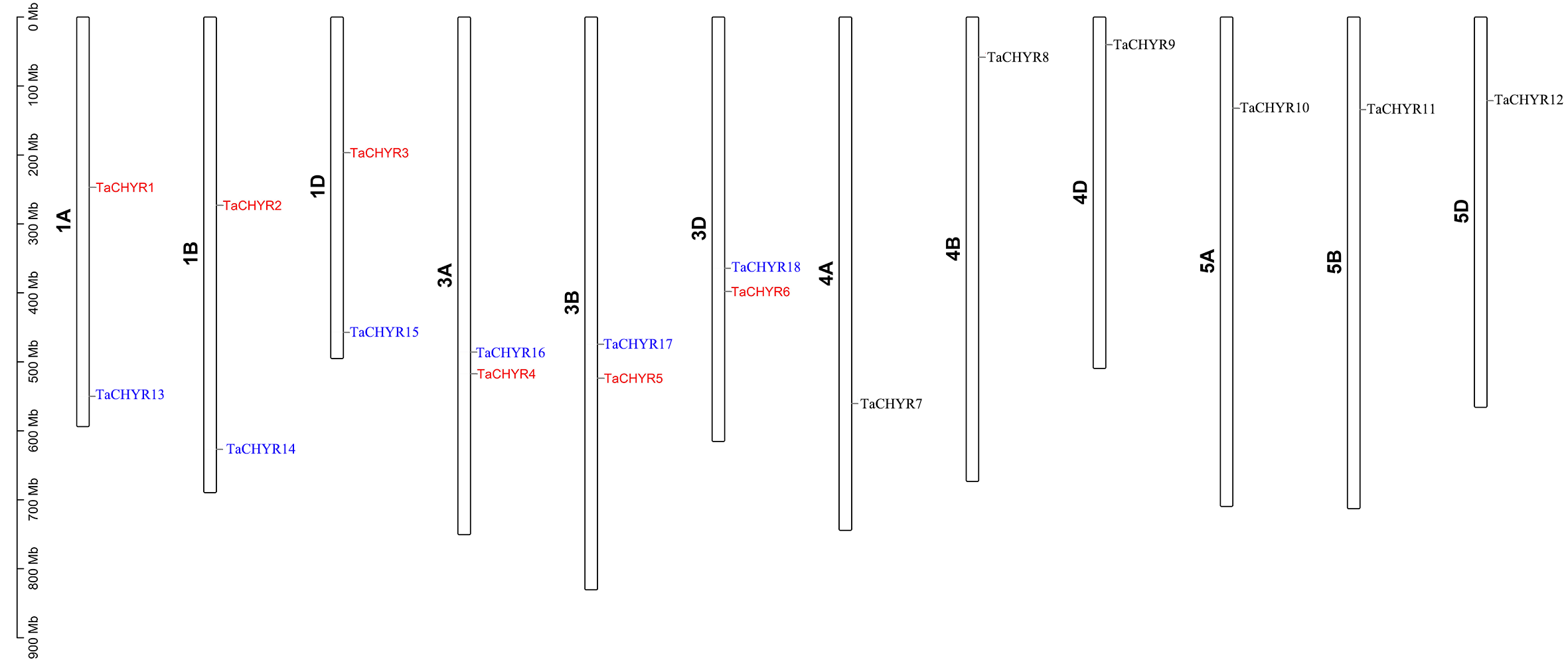

Supplement: Supplementary file 2 — Additional file 2: Fig. S2. Chromosomal localizations of TaCHYR genes in wheat. Group I, II and III members were indicated by red, black and blue, respectively. [file 12870_2022_3589_MOESM2_ESM.tif]

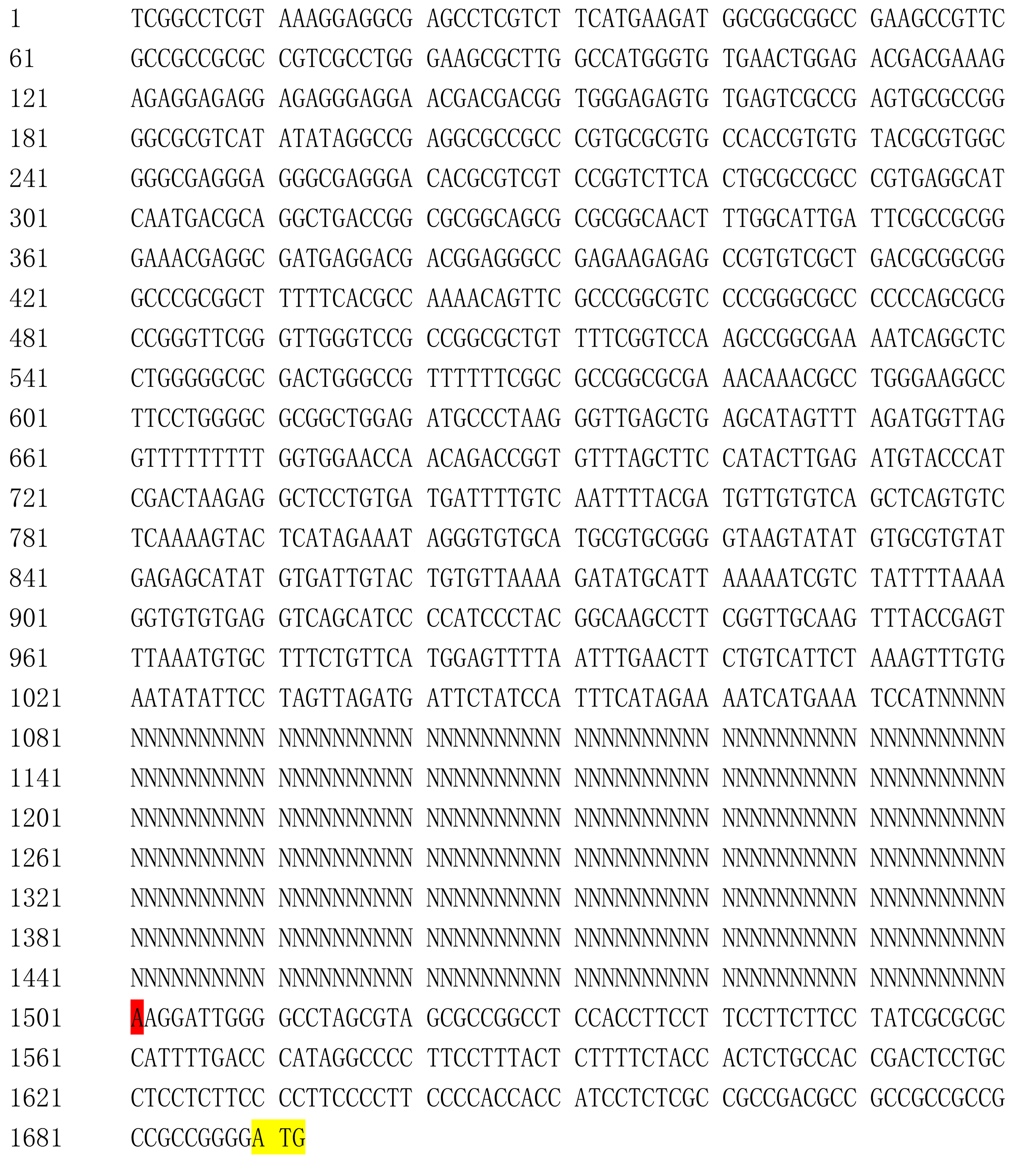

Supplement: Supplementary file 3 — Additional file 3: Fig. S3. The promoter sequences of TaCHYR11. The transcription start site (TSS) and start codon were indicated by red and yellow, respectively. [file 12870_2022_3589_MOESM3_ESM.tif]
